# Thermodynamics of hybrid manganese formate perovskites†

**DOI:** 10.1039/d4ra03700f

**Published:** 2024-09-16

**Authors:** Novendra Novendra, G. P. Nagabhushana, Alexandra Navrotsky

**Affiliations:** a Peter A. Rock Thermochemistry Laboratory and NEAT ORU, University of California Davis Davis CA 95616 USA Alexandra.Navrotsky@asu.edu; b Department of Chemistry, PES University Bangalore India; c School of Molecular Sciences and Center for Materials of the Universe, Arizona State University Tempe AZ 85287 USA

## Abstract

Hybrid organic–inorganic materials, both dense and porous, have gained significant attention in recent years due to their extreme tunability in terms of compositions and functional properties. A deep understanding of their intrinsic stability is crucial to accelerate the discovery of new compositions that are not only functional but also thermodynamically stable. Here, we report the first systematic experimental study of the effect of A-site cations on the thermodynamic stability of a series of hybrid manganese formate perovskites [AH]Mn[HCOO]_3_ with AH^+^ = CH_3_NH_3_^+^, (CH_3_)_2_NH_2_^+^, (CH_2_)_3_NH_2_^+^, CH(NH_2_)_2_^+^, and C(NH_2_)_3_^+^ using acid solution calorimetry. Our studies show that the thermodynamic stability among these does not directly correlate with their tolerance factors, in contrast to trends seen among inorganic perovskites. On the other hand the enthalpy of formation correlates linearly with the enthalpy of dissolution in aqueous hydrochloric acid of the corresponding A-site cation salt, suggesting that the interactions between the A-site cation and the framework, rather than geometric factors, dominate the energetics of these perovskites.

## Abstract 2

Thermodynamic stability of hybrid perovskites was found to be strongly influenced by the interaction between the A-site cation and the framework. Calorimetric measurements were able to estimate the relative strength of this interaction.

## Graphical abstract

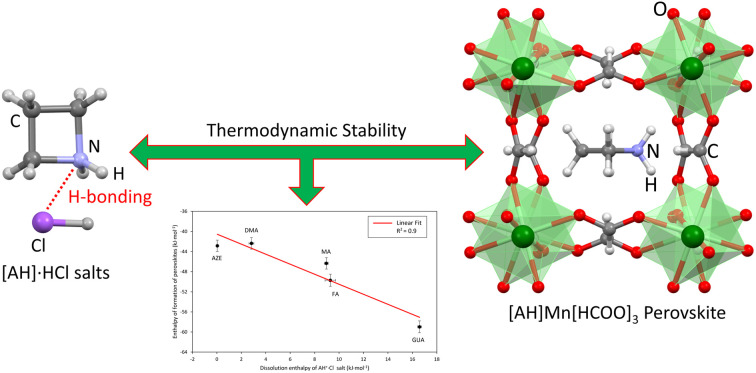

## Introduction

The term perovskite has been accepted for a wide class of materials that have a similar structure to the mineral CaTiO_3_. This structure has a general formula ABX_3_, where A is a cation located at the cavities of the framework constructed by the B cation and X anions, typically halogens or oxygen.^1^ Hybrid organic–inorganic perovskites are a subset of the perovskite family in which the cation in the A-site and/or the anion in the X-site is replaced by organic amine cations and/or organic linkers respectively. The introduction of organic components to this structure provides new functionalities and structural flexibilities previously unattainable with inorganic perovskites.^2–4^ The introduction of an organic moiety, such as formate (HCOO^−^), on the X-site leads to a compact arrangement of atoms in the structure, which is referred to as a non-porous or dense metal–organic framework.

The first discovered and characterized hybrid perovskites, cubic methylammonium lead halides (MAPbX_3_, X = Cl, Br or I)^5,6^ gained massive attention in the scientific community due to their promising properties, including high optical absorption, benign grain boundaries, high carrier mobility, and narrow bandgap. All these properties together make them an ideal candidate for application in high performance photovoltaics. Since then, several classes of hybrid perovskites have been synthesized with different organic amine cations in the A-site, such as methylammonium (CH_3_NH_3_^+^), formamidinium (CH(NH_2_)_2_^+^), and dimethylammonium ((CH_3_)_2_NH_2_^+^), and molecular linkers on the X-site, such as azide (N_3_^−^), cyanide (CN^−^), formate (HCOO^−^) and borohydride (BH_4_^−^).^2,7–10^

**Fig. 1:**
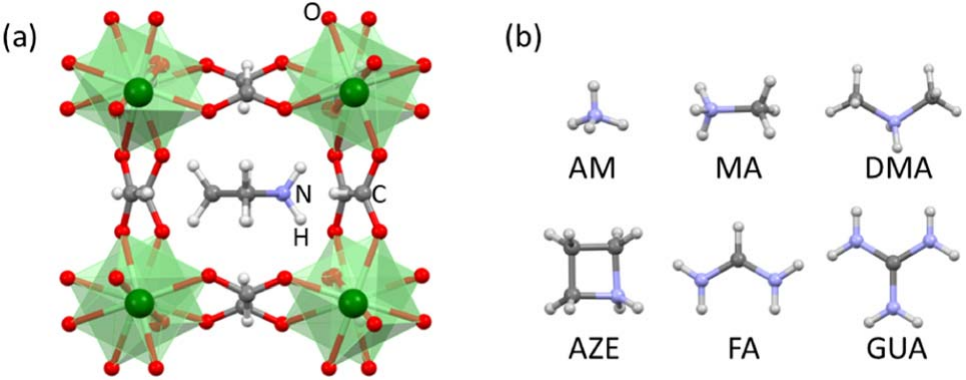
(a) Perovskite structure of AH^+^-Mn-F. (b) AH^+^ cations studied in this work: ammonium (AM, NH_4_^+^), methylammonium (MA, CH_3_NH_3_^+^), dimethylammonium (DMA, (CH_3_)_2_NH_2_^+^), azetidinium (AZE, (CH_2_)_3_NH_2_^+^), formamidinium (FA, CH(NH_2_)_2_^+^), and guanidinium (GUA, C(NH_2_)_3_^+^). Color scheme: Mn is green, O is red, N is purple, C is gray, and H is white.

A number of thermodynamic studies have been performed on hybrid perovskites, both experimentally and theoretically, to understand their stability and the effect of their compositions on the overall stability.^11–17^ However, the effect of organic A-site cations has not been explored systematically despite the importance of this substitution in controlling electronic and mechanical properties.^18–22^ In the present study, the influence of the A-site cation on the energetic stability of the hybrid perovskite is studied systematically using room temperature isothermal acid (HCl) solution calorimetry. We measure the enthalpies of dissolution of a series of manganese formate perovskites, [AH]Mn[HCOO]_3_ (AH^+^-Mn-F for short) (Fig. 1) and calculate their enthalpy of formation from their corresponding chloride salts and formic acid. This class of materials exhibit interesting functional properties, such as ferromagnetism, ferroelectricity, ferroelasticity and multiferroicity.^23–27^ The presence of formate anions allows the incorporation of a wide array of AH^+^ cations into the structure.

Here we report the synthesis, characterization, and measurement of formation energies of different AH^+^-Mn-F perovskites where AH^+^ = ammonium (AM, NH_4_^+^), methylammonium (MA, CH_3_NH_3_^+^), dimethylammonium (DMA, (CH_3_)_2_NH_2_^+^), azetidinium (AZE, (CH_2_)_3_NH_2_^+^), formamidinium (FA, CH(NH_2_)_2_^+^), and guanidinium (GUA, C(NH_2_)_3_^+^). The systematics in the resulting energetics are discussed to understand their thermodynamic stability trends.

## Experimental methods

### Synthesis

All chemicals used for synthesis were commercially available and used without further purification. We previously reported the synthesis, characterization, and calorimetric measurements of DMA-Mn-F,^14^ and the data reported is used here for comparisons. All the other [AH]Mn[HCOO]_3_ samples were synthesized using the methods reported earlier and briefly described below.

AM-Mn-F was synthesized by the reaction of MnCl_2_·4H_2_O solution in methanol with ammonium hydroxide and formic acid solutions in methanol.^28^ The covered mixed solution was left undisturbed for the crystallization process to occur. The crystals were obtained after 24 h and washed with methanol.

MA-Mn-F and AZE-Mn-F samples were synthesized by a solution diffusion method.^29^ In a typical experiment, a methanol solution of 0.5 M formic acid and methylamine or azetidine was placed at the bottom of a glass tube. Methanol was layered on top of this solution carefully and 0.1 M MnCl_2_ solution in methanol was placed carefully on top of it. The tube was sealed and left undisturbed. The crystals were collected after a week and washed with methanol.

FA-Mn-F was prepared by a solvothermal method.^14^ MnCl_2_·4H_2_O was dissolved in a mixture of ethanol–water and added to formic acid and formamide. The resultant solution was sealed and kept undisturbed at 130 °C. Formation of crystals were observed after a day and collected after two days and washed with formamide. This synthesis method is an adaptation of the previously reported synthesis of DMA-Mn-F.

GUA-Mn-F was prepared by mixing aqueous Mn(ClO_4_)_2_·6H_2_O with aqueous solution of formic acid and [C(NH_2_)_3_]_2_CO_3_. The mixture was left undisturbed to evaporate. The crystals were collected after three days and washed with ethanol.^30^

### Characterization

Powder X-ray diffraction (PXRD) patterns were recorded at room temperature by a Bruker-AXS D8 Advance diffractometer using Cu Kα radiation (*λ* = 1.54060 Å) operated at 40 kV and 40 mA. The scans were collected in the range of 10 to 70° 2*θ* with a step size of 0.02° by using a zero-background sample holder. The obtained PXRD patterns were analyzed with a whole profile fitting (WPF) technique using a Jade 6.5 software. Data refinements were performed using published crystal structures. Thermogravimetric analysis (TGA) was performed using a Netzsch 449 TG/DSC system. The measurement was done in a platinum crucible between 30–600 °C with a heating rate of 10 °C min^−1^ under a mixture of flowing argon and oxygen (40 ml min^−1^ flow rate) to mimic air but exclude CO_2_. Fourier transform infrared spectroscopy (FTIR) was done with a Bruker Equinox 55 system in the range of 400–4000 cm^−1^.

### Isothermal acid solution calorimetry

Room temperature solution calorimetry measurement was done using a CSC (Calorimetry Sciences Corporation) 4400 isothermal microcalorimeter at 25 °C. The data collection and device operation were done by using IMC data acquisition software. In each experiment, typically 15 mg of the sample was pressed to form a pellet and dropped into a Teflon cell in the calorimeter, filled with 25.0 g of 5 M HCl solvent. All weight measurements were done using a Mettler microbalance with an accuracy of 10 μg. The solvent was isothermally equilibrated for at least three hours under mechanical stirring before the introduction of the sample, and the sample was allowed to dissolve in the cell for at least three hours, ensuring the return of signal back to its initial baseline position. After each experiment, the cell was reassembled with fresh solvent.

The calorimetric curves were integrated and converted to joules using a calibration factor, based on the dissolution of KCl in water, to obtain the total heat effect due to the dissolution of the sample. For each sample, at least 4 measurements were performed, and the average was reported as the final value. The uncertainties given represent the 95% confidence interval. This experimental method is the same as in our earlier study of metal–organic frameworks where the methodology is described in detail.^14,31,32^

## Results and discussion

**Fig. 2:**
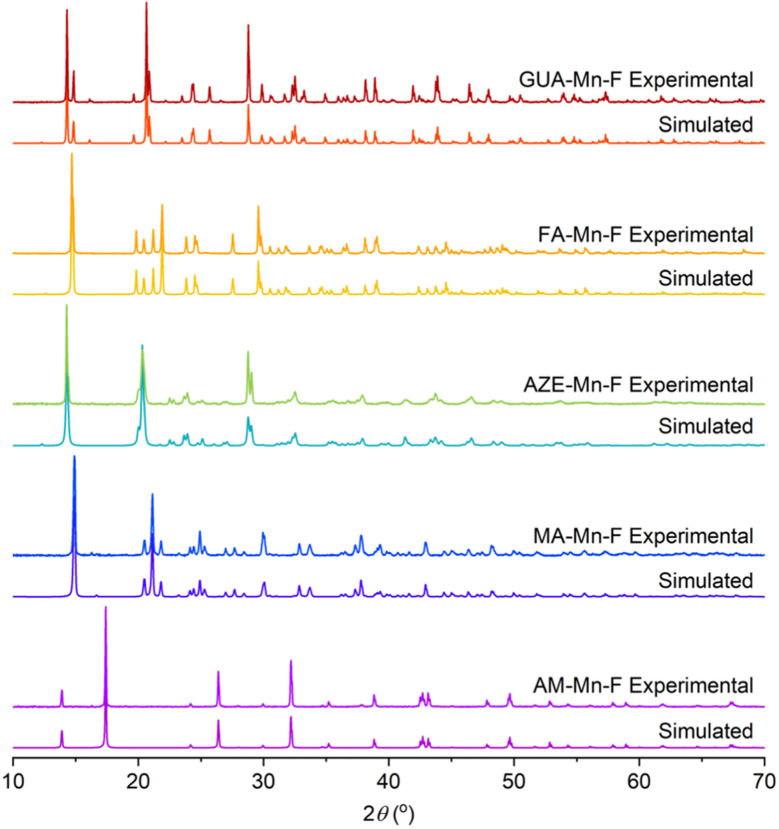
PXRD patterns for all synthesized AH^+^-Mn-F samples and their respective simulated patterns.

The characterization of AM-Mn-F, MA-Mn-F, AZE-Mn-F, FA-Mn-F, and GUA-Mn-F is discussed in detail here, while the results for DMA-Mn-F were previously reported.^14^ The PXRD patterns of all AH^+^-Mn-F samples are given in Fig. 2. All of the samples except AM-Mn-F exhibit perovskite structure with a face-centered cubic framework of Mn[HCOO]_3_^−^ and the AH^+^ cation that occupies the cavity, while the AM-Mn-F sample crystallizes in hexagonal structure. Whole pattern fitting using the published crystal structures of each sample was performed using MDI Jade 6.5 software. The results confirmed the formation of single phases, and the corresponding lattice parameters are tabulated in Table 1.

**Table 1:** Lattice parameters of the AH^+^-Mn-F samples

Sample	Space group	*a* (Å)	*b* (Å)	*c* (Å)	*R* ^2^ value (%)
AM-Mn-F	*P*6_3_22	7.362	7.362	8.484	5.37
MA-Mn-F	*Pnma*	8.684	11.943	8.161	3.35
AZE-Mn-F	*Pnma*	8.681	12.313	8.882	5.86
FA-Mn-F	*C*2/*c*	13.819	8.702	8.477	4.21
GUA-Mn-F	*Pnna*	8.524	11.980	9.058	6.35

FTIR spectra of all samples and the band assignments are given in Fig. S1.† The purity of the samples was confirmed by the absence of the peaks corresponding to the starting materials in the FTIR spectra.

**Fig. 3:**
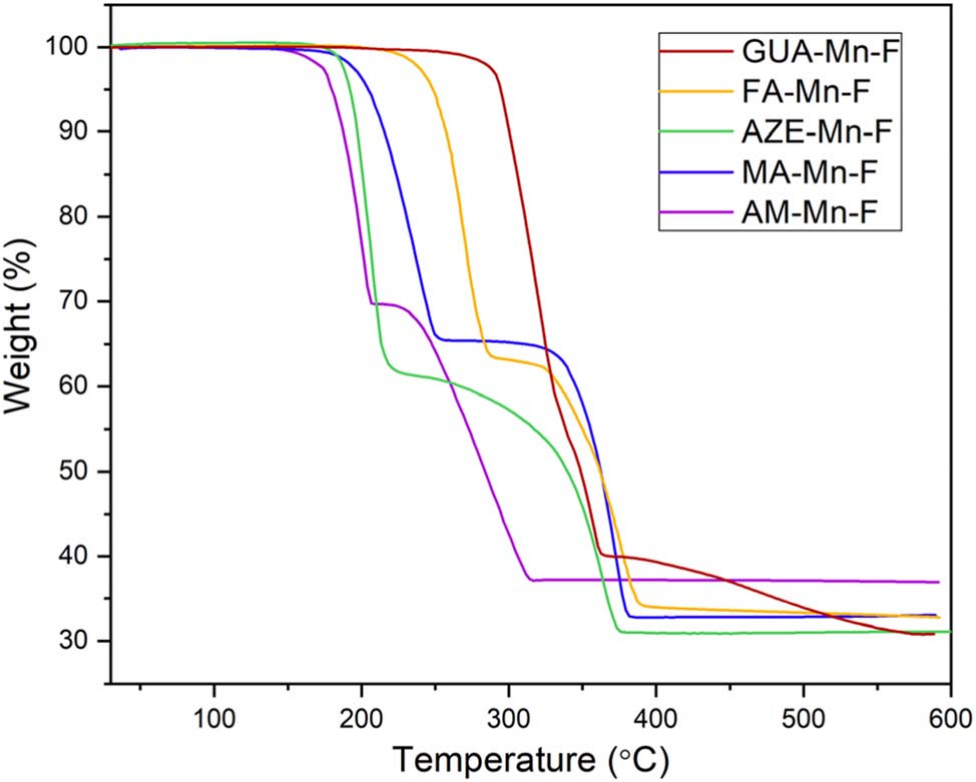
TGA curves of all AH^+^-Mn-F samples.

Thermal stability of the samples was investigated using thermogravimetric analysis (TGA) and the TGA curves of all the samples are given in Fig. 3. Four of the samples, AM-Mn-F, MA-Mn-F, AZE-Mn-F and FA-Mn-F showed a distinct two-step mass loss, while GUA-Mn-F showed more than two-step mass loss. GUA-Mn-F exhibits a significantly higher decomposition temperature of 292 °C compared to the other samples, *viz.* FA-Mn-F (250 °C), MA-Mn-F (204 °C), AZE-Mn-F (192 °C), and AM-Mn-F (182 °C). For all samples, the first step corresponds to the loss of one formate and one amine per formula unit, which results in the formation of Mn[HCOO]_2_. The experimental and calculated stoichiometric (in parentheses) weight loss percentages are 30.33 (29.83), 34.46 (34.26), 39.26 (41.16), 37.11 (38.32), and 40.86(41.62) for AM-Mn-F, MA-Mn-F, AZE-Mn-F, FA-Mn-F and GUA-Mn-F respectively. The second mass loss step corresponds to the decomposition of Mn[HCOO]_2_ to the final form of binary manganese oxides (confirmed by PXRD analysis of the residue). The total experimental weight loss percentages are 63.09, 66.97, 68.91, and 67.24 for AM-Mn-F, MA-Mn-F, AZE-Mn-F and FA-Mn-F respectively. In the case of GUA-Mn-F, the subsequent mass loss step might include a more complex combination of two or more steps together,to the decomposition of Mn[HCOO]_2_ to the final form of binary manganese oxides. The total experimental weight loss for GUA-Mn-F is 69.17%.

To assess the thermodynamic stability of AH^+^-Mn-F samples, room temperature isothermal acid solution calorimetry was performed. The dissolution enthalpy (Δ*H*_ds_) of the samples and their components in 5.0 M HCl were collected and used to calculate the enthalpy of formation (Δ*H*_f_) of the AH^+^-Mn-F samples using thermochemical cycle in Table 2. The measured Δ*H*_ds_, calculated Δ*H*_f_ of AH^+^-Mn-F samples and other thermochemical data needed for the calculation are summarized in Table 3.

**Table 2:** Thermochemical cycle used to calculate the formation enthalpy of AH^+^-Mn-F^a^

Reactions	Enthalpy (Δ*H*)
[AH]Mn(HCOO)_3(s)_ → Mn_(aq.)_^2+^ + AH_(aq.)_^+^ + 3HCOO_(aq.)_^−^	Δ*H*_1_
MnCl_2(s)_ + 2H^+^ → Mn_(aq.)_^2+^ + 2HCl_(5M HCl,aq.)_	Δ*H*_2_
[AH]·Cl_(s)_ + H_(aq.)_^+^ → AH_(aq.)_^+^ + HCl_(5M HCl,aq.)_	Δ*H*_3_
HCOOH·*x*H_2_O_(l)_ → HCOO_(aq.)_^−^ + H_(aq.)_^+^ + *x*H_2_O_(aq.)_	Δ*H*_4_
*x*H_2_O_(l)_ → *x*H_2_O_(aq.)_	Δ*H*_5_
MnCl_2(s)_ + [AH]·Cl_(s)_ + 3HCOOH·*x*H_2_O_(l)_ → [AH]Mn(HCOO)_3(s)_ + 3HCl_(5M HCl,aq.)_ + 3*x*H_2_O_(l)_	Δ*H*_6_ = Δ*H*_f,_ = −ΔH_1_ + Δ*H*_2_ + Δ*H*_3_ +3Δ*H*_4_ − 3Δ*H*_5_

a *x* = 0.07670 per formula unit.

**Table 3:** Thermochemical data used for the calculation of enthalpy of formation of AH^+^-Mn-F (AH^+^ = AM, MA, DMA, AZE, FA, and GUA)

Compound	Δ*H*_ds_^a^ (kJ mol^−1^)	Δ*H*_f_ (kJ mol^−1^)
HCOOH	−0.84 ± 0.17 (4)^b^	
H_2_O	−0.35^b^	
MnCl_2_	−48.06 ± 1.01 (4)^b^	
AM·Cl	17.99 ± 0.16 (4)	
MA·Cl	8.96 ± 0.15 (5)	
DMA·Cl	2.83 ± 0.20 (6)	
AZE·Cl	0.04 ± 0.01 (5)	
FA·Cl	9.29 ± 0.44 (7)	
GUA·Cl	16.58 ± 0.16 (4)	
AM-Mn-F	−10.32 ± 0.09 (4)	−22.20 ± 1.15
MA-Mn-F	4.73 ± 0.05 (5)	−46.35 ± 1.14
DMA-Mn-F	−5.29 ± 0.06 (4)^b^	−42.38 ± 1.15^b^
AZE-Mn-F	−7.68 ± 0.09 (4)	−42.86 ± 1.14
FA-Mn-F	8.43 ± 0.12 (7)	−49.72 ± 1.22
GUA-Mn-F	24.97 ± 0.29 (6)	−58.97 ± 1.18

a Values given in parentheses are the number of experiments performed. The uncertainties given in the enthalpy of formation represent a 95% confidence interval.

b Ref. 14, 32 and 33.

The calculated enthalpy of formation, Δ*H*_f,_ for all the AH^+^-Mn-F samples from their corresponding chlorides and formic acid are exothermic indicating that they are thermodynamically stable at room temperature and that the formation reaction is thermodynamically driven. Amongst the series of samples, AM-Mn-F shows the least exothermic enthalpy of formation, −22.20 ± 1.15 kJ mol^−1^. As mentioned previously, AM-Mn-F, with NH_4_^+^ cation is the only sample that does not have a perovskite structure.

Among all the samples with perovskite structures, GUA-Mn-F has the most exothermic enthalpy of formation of −58.97 ± 1.18 kJ mol^−1^, indicating that it is the most thermodynamically stable compound in this series, followed by FA-Mn-F, MA-Mn-F, AZE-Mn-F, and DMA-Mn-F. The enthalpies of formation of all the perovskites excluding GUA-Mn-F from their binary components are rather similar, ranging between −42 to −50 kJ mol^−1^. These strongly exothermic enthalpies suggest that entropy effects, which are expected to be small, will not change the trends in free energy significantly from those seen in enthalpy.

To further understand the role of the AH^+^ cations in the overall stability of the structure, we examine several factors including tolerance factor and the hydrogen bonding abilities of the AH^+^ cations. The Goldschmidt tolerance factor is used extensively to explain the stability of perovskites, especially among inorganic perovskites.^34,35^ This parameter explains the degree of distortion of the structure from an ideal cubic perovskite structure. The value is calculated from the ratio of the ionic radii of the cations and the anion and is given by:1
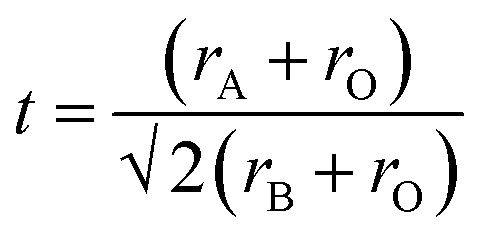
where *r*_A_, *r*_B_, and *r*_O_ correspond to the ionic radii of the cations and the anion (oxygen) of ABO_3_ perovskite. The ideal perovskite structure will have a tolerance factor value of 1. The application of the Goldschmidt tolerance factor to inorganic perovskites is straightforward due to the availability of ionic radii data, while the presence of molecular cations and anions in hybrid perovskite introduces a layer of complexity to the calculation. It is difficult to define the ionic radius of a molecular ion due to the presence of hydrogen bonding that causes the bond lengths to vary. Kieslich *et al.*^36,37^ tried to estimate the effective radii of the organic ions by using available crystallographic data of hybrid perovskites to overcome this problem. Additionally, the organic cation was assumed to be a rigid sphere having free rotational freedom around the center of mass, while the ions that have high anisotropy (such as HCOO^−^) were assumed to be rigid cylinders. With this model, the effective ionic radius (or effective ion height for the anisotropic ion case) was defined as the sum of the distance between the center of mass of the ion to the farthest atom from the center of mass, excluding hydrogen atoms, and the ionic radius of that atom. This is given by:

2 *r*_A,eff_ = *r*_mass_ + *r*_ion_

And the modified tolerance factor equation is given by:3
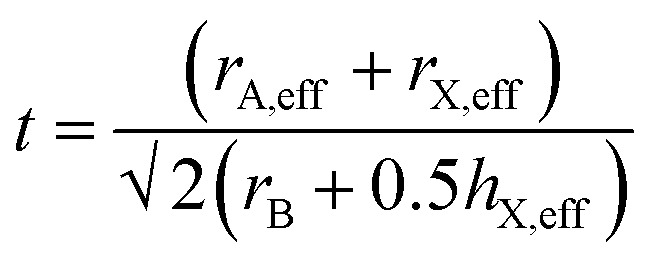


For formate, the effective radius *r*_X,eff_ = 136 pm while the effective height *h*_X,eff_ = 447 pm. The effective radius of the AH^+^ cations and the calculated tolerance factor for each structure is summarized in Table 4.

**Table 4:** Size of AH^+^ cations and the calculated tolerance factor of AH^+^-Mn-F samples

	AH^+^ cation size^a^ (pm)	Tolerance factor (*t*)	Δ*H*_f_ (kJ mol^−1^)
AM-Mn-F	146	0.65	−22.20 ± 1.15
MA-Mn-F	217	0.81	−46.35 ± 1.14
DMA-Mn-F	272	0.94	−42.38 ± 1.15^a^
AZE-Mn-F	250	0.89	−42.86 ± 1.14
FA-Mn-F	253	0.90	−49.72 ± 1.22
GUA-Mn-F	278	0.96	−58.97 ± 1.18

a Ref. 14 and 37.

The calculated tolerance factors show that the modified equation at large produced a result that agrees with the trend seen among inorganic perovskites. The samples that have perovskite structure show tolerance factors ranging between 0.81 and 0.96, which are in the normal range to form a perovskite structure. AM-Mn-F, which has a non-perovskite structure, has a tolerance factor of 0.65, which is lower than the commonly accepted limit to form a perovskite structure (<0.8).^38^

**Fig. 4:**
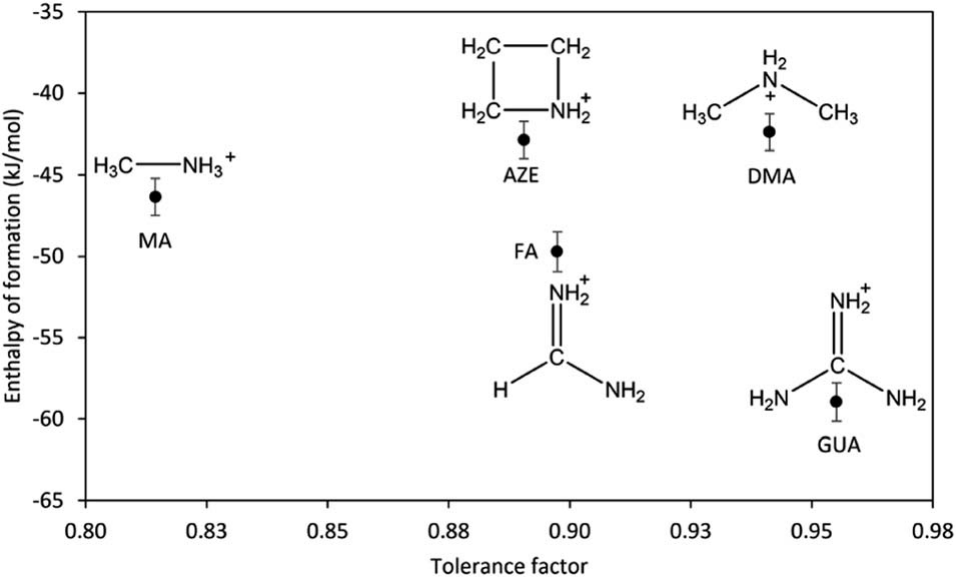
Enthalpy of formation of AH^+^-Mn-F (AH^+^ = MA, DMA, AZE, FA, and GUA) as a function of tolerance factor.

To be able to properly assess the effect of the AH^+^ cations on the overall stability and to rule out the contribution of structural changes to the stability, we consider only the samples with perovskite structure including DMA-Mn-F from the previous report.^14^ The correlation between the enthalpy of formation of AH^+^-Mn-F and the tolerance factor is shown in Fig. 4.

As seen from the figure, the change in the enthalpy of formation of AH^+^-Mn-F series as a function of tolerance factor does not exhibit any clear trend. AZE-Mn-F and FA-Mn-F, with similar cation (AH^+^) size and similar tolerance factor, show a significantly different enthalpy of formation. The same is seen with DMA-Mn-F and GUA-Mn-F. On the other hand, AZE-Mn-F with larger tolerance factor shows lower stability than MA-Mn-F, which is the opposite of what is observed in inorganic perovskites where the enthalpy of formation becomes more exothermic as the tolerance factor increases and approaches unity.^34,39–43^ This indicates that the geometric mismatch represented by the tolerance factor is not the major component that affects the resulting thermodynamic stability among hybrid perovskite. For further understanding, we consider the perovskites with AH^+^ cation that has a single carbon atom but a different number of nitrogen atoms. We observe that the enthalpy of formation becomes more exothermic as the number of nitrogen atoms increases (MA-Mn-F > FA-Mn-F > GUA-Mn-F). The same is not seen for the perovskites with AH^+^ cations that have a single nitrogen atom and a different number of carbon atoms. In fact, a reverse trend is seen here (MA-Mn-F < DMA-Mn-F ≈ AZE-Mn-F). Unlike inorganic perovskites, the cation in hybrid perovskites is a molecular cation, specifically in this case a protonated amine. In addition to the electrostatic interaction, the presence of N–H and C–H moieties in the cation structure provides significant hydrogen bonding between the cation in the cavity and the oxygen atom in the framework of the perovskite, having both strong N–H⋯O hydrogen bonds and weak C–H⋯O hydrogen bonds.^18,44^ Due to the different structures and compositions of the AH^+^ cations, one expects that the hydrogen bonding interaction between the cation and the framework would affect the thermodynamic stability of the hybrid perovskites under study.

Here, a convenient way to estimate the relative strength of the hydrogen bond is proposed by means of the calorimetric measurements of the AH^+^·Cl salts. From the available crystal structure of AH^+^·Cl salts, the chlorine atoms and the AH^+^ groups are linked together by hydrogen bonds.^45–47^ The enthalpy associated with the dissolution reaction of AH^+^·Cl in HCl is shown below:

4 [AH]·Cl_(s)_ + H^+^_(aq.)_ → AH^+^_(aq.)_ + HCl_(5M HCl,aq.)_

**Fig. 5:**
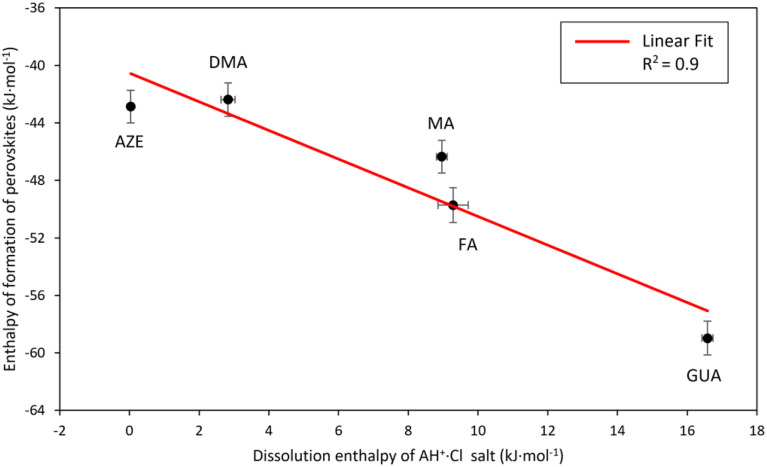
Enthalpy of formation of AH^+^-Mn-F (AH^+^ = MA, DMA, AZE, FA, and GUA) as a function of drop solution enthalpy of AH^+^·Cl salt.

The dissolution enthalpy would be proportional to the enthalpy required to break the hydrogen bonds between the AH^+^ groups and the Cl atoms in the salt structure. One would expect that the relative strength of the hydrogen bonding in AH^+^·Cl salts would also translate to the relative strength of the hydrogen bonding between the AH^+^ cations and the surrounding frameworks in the AH^+^-Mn-F perovskites. Following this consideration, the enthalpy of formation of the perovskites as a function of dissolution enthalpies of corresponding AH^+^·Cl is plotted in Fig. 5.

Fig. 5 shows that there is a strong linear correlation between the perovskite enthalpy of formation and the dissolution enthalpy of the cation salts in 5N HCl. As the dissolution enthalpy of the cation salts becomes more endothermic, the corresponding perovskite enthalpy of formation from binary constituents becomes more exothermic. This seems to be very intuitive in that the harder it is to protonate the salt to form the cation, the stronger would be the hydrogen bonding between the AH^+^ groups and Cl atoms. Consequently, this would result in stronger hydrogen bonding between AH^+^ cations and the surrounding frameworks of the perovskite structure. Our correlation also agrees with the computational results reported in the literature for the relative magnitude of hydrogen bonding energies between the cations and the perovskite framework for DMA, AZE, and GUA-Mn-F, where it was found that the hydrogen bonding energy is the strongest for GUA-Mn-F, followed by DMA-Mn-F and AZE-Mn-F.^18,44^

This correlation between the formation enthalpies of the hybrid perovskite and the hydrogen bonding energies of the AH^+^ cation provides an opportunity to predict the stability of other hybrid perovskites based on the hydrogen bonding ability of the A-site cation. For example, here the slope of the observed linear correlation between the perovskite enthalpy of formation and the dissolution enthalpy of the cation salts is 1 kJ mol^−1^, indicating that the perovskite stability would change by 1 kJ mol^−1^ per unit of cation salt dissolution enthalpy. Extending this result to other hybrid perovskite systems with a molecular A-site cation would facilitate in prediction of the stability of the perovskite structures just by knowing the dissolution enthalpy of the A-site cation salts and thus would identify the most viable sample compositions for the synthesis. Furthermore, our experimental results are in alignment with the earlier DFT calculations by Tenuta and coworkers where they showed that the limited solubility of decomposition reaction products (AH^+^·Cl salt) is anticipated to improve the thermodynamic stability of the hybrid perovskites.^15^

## Conclusions

We present a systematic study of the effect of A-site cation substitution on the thermodynamic stability of AH^+^-Mn-F (AH^+^ = AM, MA, DMA, AZE, FA, and GUA), a hybrid organic–inorganic perovskite system using room temperature isothermal acid solution calorimetry. The simple inorganic NH_4_^+^ cation resulted in the formation of a non-perovskite structure with the least thermodynamic stability in the series. Among the other AH^+^-Mn-F perovskites, GUA-Mn-F perovskite was the most stable followed by FA-Mn-F, MA-Mn-F, and AZE-Mn-F & DMA-Mn-F with similar stability. Unlike inorganic perovskites, the stability of hybrid perovskite was found to be governed more by the interaction between the A-site cation and the framework, mostly through the hydrogen bonding, than by the tolerance factor which reflects the degree of size mismatch and possible distortion of the structure. We showed that the relative strength of this hydrogen bonding energy can be estimated by a simple dissolution enthalpy measurement of the cation salts and this dissolution enthalpy correlates linearly with the enthalpy of formation of the perovskites. In the vast number of different possible composition combinations among hybrid perovskite systems, this relationship provides a very useful tool to predict the stability and accelerate the discovery of samples that are feasible for experimental synthesis and real-world applications.

## Data availability

The data supporting this article have been included as part of the ESI.†

## Conflicts of interest

There are no conflicts to declare.

## Supplementary Material

RA-014-D4RA03700F-s001
