# Supplementary material for: Thermodynamics of hybrid manganese formate perovskites
Source: RSC Adv. 2024 Sep 16;14(40):29301–7. doi: 10.1039/d4ra03700f (PMC11403386; doi:10.1039/d4ra03700f)
Supplement: RA-014-D4RA03700F-s001 [file RA-014-D4RA03700F-s001.pdf]

Supplementary Information (SI) for RSC Advances.
This journal is © The Royal Society of Chemistry 2024

Electronic Supplementary Information

Thermodynamics of Hybrid Manganese Formate Perovskites

Novendra Novendra[a], G.P. Nagabhushana[a,b], and Alexandra Navrotsky[a,c]

[a] *Peter A. Rock Thermochemistry Laboratory and NEAT ORU, University of California Davis, Davis CA 95616 USA*
[b] *Department of Chemistry, PES University, Bangalore, India*
[c] *School of Molecular Sciences and Center for Materials of the Universe, Arizona State University, Tempe AZ 85287 USA*

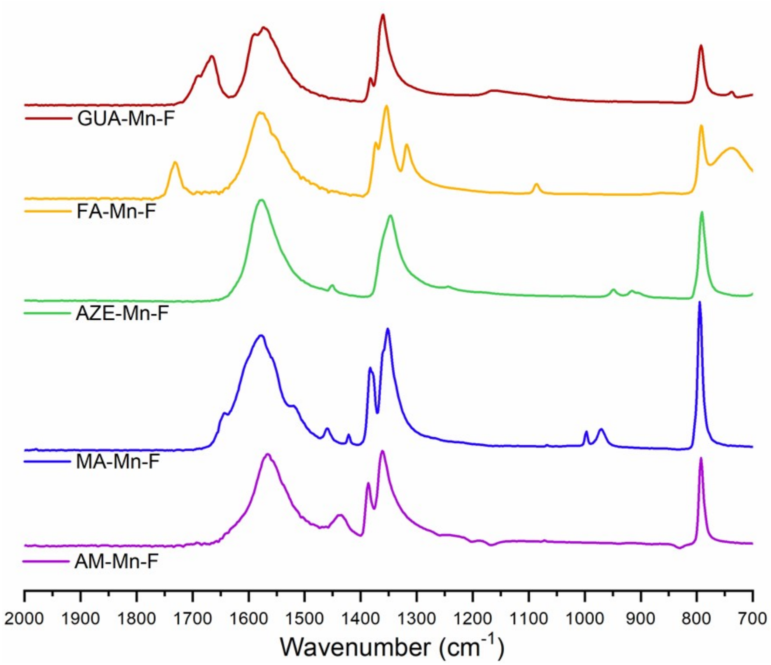

Fig. S1 FTIR patterns of all AH^+-Mn-F samples

Table S1 IR absorption bands (cm^{-1}) and their assignments for all AH^+-Mn-F samples

**Assignments**	**AM-Mn-F**	**MA-Mn-F**	**AZE-Mn-F**	**FA-Mn-F**	**GUA-Mn-F**
$\delta_{as}(NH_2)$				1732 (s)	1689 (sh)
		1640 (sh)			1666 (s)
ν_4 OCO stretch, asym	1566 (s)	1577 (s)	1576 (s)	1580 (s)	1589 (sh)
		1520 (sh)			1574 (s)
ν(-CH_2-)		1460 (w)	1450 (w)		
$\nu_4(NH_4+)$	1435 (m)				
ν_5 OCO deformation, asym	1387 (s)	1382 (s)		1373 (sh)	1382 (m)
ν_2 OCO stretch, sym	1362 (s)	1352 (s)	1346 (s)	1354 (s)	1360 (s)
$\rho(NH_2)$				1317 (s)	
ν(C–N)			1245 (vw)		1163 (w)
$\rho(NH_2)$				1086 (s)	
ν_6 out-of-plane deformation		970 (w)	950 (w)		
			916 (w)		
ν_3 OCO deformation, sym	793 (s)	795 (s)	791 (s)	793 (s)	793 (s)
$\omega(NH_2)$				736 (b)	
$\rho_r(NH_2)$					737 (w)
